# Evaluation of four learning collaboratives for improving diagnostic excellence in radiology

**DOI:** 10.1002/lrh2.70035

**Published:** 2025-08-22

**Authors:** Laura M. Holdsworth, Heather Z. Mui, Kandice Garcia Tomkins, Kay Zacharias‐Andrews, Mythreyi Bhargavan‐Chatfield, Marcy Winget, David Larson

**Affiliations:** ^1^ Division of Primary Care and Population Health, Department of Medicine Stanford University School of Medicine Palo Alto California USA; ^2^ Department of Radiology Stanford University School of Medicine Stanford California USA; ^3^ American College of Radiology Reston Virginia USA

**Keywords:** learning collaborative, qualitative methods, quality improvement, radiology

## Abstract

**Introduction:**

Learning collaboratives are frequently used within healthcare to facilitate practice improvement through collaboration among clinical teams across multiple organizations. The aim of this study was to use the Agency for Healthcare Research and Quality (AHRQ) collaborative taxonomy to identify collaborative elements that influence successful implementation of clinical practices and sustain improvements in four radiology learning collaboratives.

**Methods:**

We used an ethnographic approach to evaluate a learning collaborative network using the AHRQ collaborative taxonomy. Data collection included observations, interviews, and review of site performance metrics.

**Results:**

We identified four themes that spanned the four AHRQ taxonomy primary elements (innovation, time, communication, social system) that explained the influence of the collaborative structure on site improvements and sustained success: (1) structured education in quality improvement and access to quality improvement tools provides a framework for quality improvement; (2) an expert‐guided, structured improvement process sets the pace of improvement; (3) intentional participant interaction and contribution in meetings reinforces accountability; and (4) credible leadership and facilitation sustains participation.

**Conclusions:**

While we identified all four primary elements of the AHRQ framework as important for a successful learning collaborative, social system elements were particularly dominant in their influence on sites' success. In particular, expert, credible leaders who provided the right tools, at the right time and pace, with constructive guidance were critical for maintaining site engagement and driving problem‐solving.

## 1 INTRODUCTION

Learning collaboratives are structured, time‐limited systems that facilitate practice improvements in healthcare by promoting collaboration among clinical teams across multiple organizations.^1^ Originating from quality improvement (QI) methodologies, learning collaboratives have become essential in addressing complex healthcare challenges.^2^ Learning collaboratives employ a framework that includes regular expert‐led sessions and peer‐to‐peer sharing, allowing participants to learn from each other and implement changes in patient care practices over a defined period. The significance of learning collaboratives lies in their ability to drive measurable improvements in healthcare delivery and patient outcomes through collective learning and accountability. Several systematic reviews have documented their positive impacts, including enhanced management of chronic conditions and improved health outcomes resulting from integrated care approaches.^3^, ^4^, ^5^

The effectiveness of learning collaboratives, however, can vary based on factors such as stakeholder engagement and the context of implementation; though many studies are of low quality.^3^, ^4^, ^6^ Previous research has often focused on whether and how learning collaboratives are effective, but there has been less work on what the specific attributes or elements of collaboratives are that make them effective or not.^5^, ^7^ The Agency for Healthcare Research and Quality (AHRQ) has produced a taxonomy of learning collaborative attributes that can be used to develop learning collaboratives and assess their effectiveness.^8^ The taxonomy provides descriptions of collaborative elements, but does not indicate or hypothesize how elements individually or collectively produce change. The aim of this study was to use the AHRQ collaborative taxonomy to identify elements and explain how they interact to influence successful implementation of clinical practices and sustain improvements in four radiology learning collaboratives.

## 2 METHODS

### 2.1 Design

We took an ethnographic approach to evaluate a learning collaborative network using the AHRQ collaborative taxonomy to identify and categorize elements of the collaborative structure that influenced successful implementation and sustained improvements. The Stanford University Institutional Review Board determined that this was a program evaluation and exempted it from human subjects research review.

### 2.2 Setting and intervention description

**FIGURE 1:**
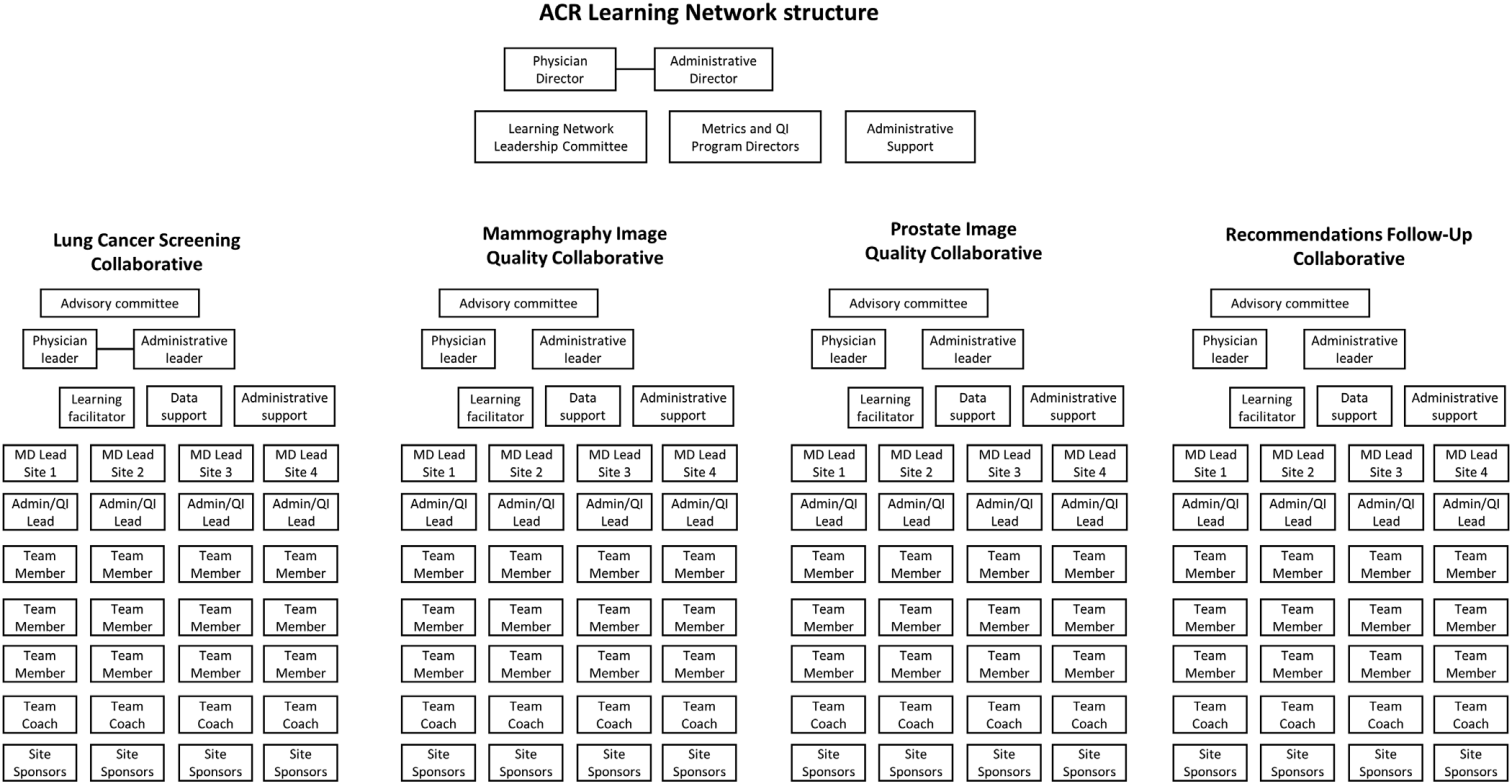
ACR learning network organizational structure. MD, medical doctor; QI, quality improvement. *Source*: Adapted from Larson et al.^9^

The learning collaborative network was run by the Quality and Safety Commission of the American College of Radiology (ACR) and included four learning collaboratives, each focused on an aspect of radiology care: lung cancer screening, mammography image quality, prostate image quality, and recommendations for follow‐up of incidental lung nodule findings. Each collaborative utilized the same organizational structure (Figure 1) and quality improvement learning collaborative design, but had different goals and participants, which allowed for a comparison of how a common collaborative structure can influence improvement efforts on different aspects of radiology care (i.e., screening volumes, image quality, recommendations follow‐up). At the time of writing, the ACR had run three cohorts of collaboratives: Cohort 1 (March–November 2022), Cohort 2 (March–September 2023), and Cohort 3 (November 2023–July 2024).

The collaborative network was designed with the first 1–2 months of each cohort focused on measurement development specific to each collaborative, and aimed to build on the work of the previous cohorts, such as agreeing on criteria for image quality or how screening volume would be measured and reported. This was followed by weekly meetings, alternating between learning sessions and collaborative‐specific “walk the wall” sessions. Learning sessions were conducted jointly across all four collaboratives, covering quality improvement concepts and materials, with small group breakout sessions for participant discussions and designated team time. Walk the wall sessions were specific to each collaborative, where participants with the same goal and outcome metric shared their progress and received feedback. The collaborative culminated in 2 days of graduation, where all participating collaborative sites shared a final presentation of their quality improvement project. All sessions were conducted virtually on Zoom. The networked structure of radiology collaboratives utilizing both shared and separate virtual learning forums, and building on learning across cohorts is unique.

### 2.3 Participants

Participating organizations in each collaborative were selected through an application process run by the ACR; the screening process has been previously described.^9^ Each organization was represented by an average of nine participants (range 5–21) and each team included the following standard roles: project sponsor, team leader, physician leader, QI coach, and team member(s).

### 2.4 Data collection

#### 2.4.1 Observations

Two researchers (HZM, LMH) observed Cohort 2 collaborative activities, including learning sessions, measurement development, and walk the walls for two of the four collaboratives (mammography and lung cancer screening) and graduations. At the beginning of the first learning session, the researchers introduced themselves as evaluators of the network and explained their role as observers of collaborative sessions. Observational notes were typed up into a narrative account for each observed session, with each researcher adding their own reflections and summary assessment.

#### 2.4.2 Interviews

After the Cohort 2 collaboratives ended, we conducted in‐depth interviews with team leaders, QI coaches, and/or team members from the mammography and lung cancer screening collaboratives to further explore participant experiences with the learning collaborative network. Participants were recruited via email directly by HZM; up to two representatives from each site were invited to participate to capture different perspectives. Interviews were conducted via Zoom by HZM and covered the following topics: site achievements, barriers and facilitators to improvement, how the collaborative supported improvement efforts, views on the learning collaborative format and structure, and future plans for sustainment. In addition, we conducted interviews with Cohort 1 participants from all four collaboratives approximately 1.5 years post‐graduation to explore longer‐term sustainability of program improvements as they related to participation in the collaborative (see Supporting Information for topic guides). Participants from all four collaboratives were recruited via email, with ACR collaborative leaders connecting past participants with HZM via email, who then scheduled and conducted the interviews. Interviews were recorded with permission and transcribed for analysis.

### 2.5 Analysis

Qualitative data were coded and analyzed using an explanatory matrix approach.^10^ Impressions from observations were used to develop an initial codebook along with the AHRQ taxonomy framework, presented in Table 1.^8^ To familiarize ourselves with the data prior to coding, researchers reviewed notes and transcripts, or listened to interview audio recordings. Observation notes and interview transcripts were then imported into NVivo and coded using an inductive and deductive approach by HZM. After all data were coded, data were organized into a matrix, with AHRQ concepts as column headings and each site's data as a row, to identify patterns to explain how collaborative elements as defined in the AHRQ taxonomy related to program success and sustainment.^10^ In order to explore AHRQ elements in relation to success in achieving goals set by sites, we included columns in the matrix that summarized quantitative data that was routinely reported to the ACR Network leaders, such as patient volumes, percent of high‐quality images, or percent of appropriate and timely follow‐up. The matrices were reviewed and discussed by two researchers (HZM, LMH) to identify patterns in the data. The resulting patterns that explain the relationship between structural collaborative elements and success are presented as themes.

**TABLE 1:** AHRQ learning collaborative taxonomy elements.^8^

Primary element	Secondary element
Innovation	Type of change
	Degree of prescription
	Scope
	Supporting tools
Communication	Mode or venue
	Directionality
	Frequency
	Degree of formality
Time	Duration of learning collaborative
	Duration of member recruitment
	Rate of attainment or adoption
	Sustainability of learning collaborative
Social systems	Degree of credibility of host or convener and leadership
	Membership characteristics
	Governance
	Purpose and degree of shared vision
	Culture of learning collaborative
	Members activity level
	Roles, process, and structure

## 3 RESULTS

We observed 39 weekly collaborative sessions between March 2023 and September 2023. We conducted 20 interviews with Cohort 2 participants, representing 12 sites (7 mammography, 5 lung cancer screening), between October 19, 2023, and December 1, 2023. We conducted 12 interviews with Cohort 1 participants across all four collaboratives between June 2024 and August 2024. Interviews lasted a median length of 57 minutes (range 30–82 min). Interview participant characteristics are presented in Table 2.

**TABLE 2:** Interview participant characteristics.

Characteristic	*N* = 32
Cohort	
Cohort 1	12
Cohort 2	20
Collaborative	
Mammography imaging	15
Lung cancer screening	13
Prostate imaging	2
Recommendations follow‐up	2
Region	
Northeast	11
Southwest	8
West	6
Southeast	4
Midwest	3
Role	
QI coach	16
Team leader	13
Team member	3

Table 3 shows information about the number of sites who completed participation in their respective collaborative (i.e., graduated) and achieved their performance goal by graduation. Overall, only one site in cohort two dropped out, indicating high overall retention in the collaborative. Half of all participating sites achieved their goal by graduation; notably, none of the lung cancer screening sites had achieved goal at graduation, though participants stated that the length of the collaborative (6–9 months) was insufficient time for improvement efforts to be reflected in the data. Of the 32 interviewed participants, 12 were from a site that had achieved their goal at graduation, 13 were from a site that was improving, but had not achieved goal, and 7 had not made notable improvement. For our analysis, we focused on what participants felt helped them either achieve success, or moved them in that direction.

**TABLE 3:** Number of sites that graduated from their collaborative and achieved performance goal by cohort.

	Cohort 1	Cohort 2	Cohort 1 + 2
Sites who completed participation in the collaborative (i.e., graduated)			
Lung cancer screening	6	5	11
Mammography imaging	6	7 (of 8)^a^	13
Prostate imaging	5	4	9
Recommendations follow‐up (RFU)	4	3	7
**Total**	**21**	**19**	**40**
Sites who achieved performance goal^b^ at graduation			
Lung cancer screening	0	0	0
Mammography imaging	4	4	8
Prostate imaging	4	3	7
RFU: appropriateness	2	2	4
RFU: timely follow‐up	2	1	3
**Total** ^c^	**11**	**9**	**20**

^a^ One site in the mammography cohort 2 dropped out.

^b^ Goals for each collaborative were defined as follows: lung cancer screening: increase the average number of patients screened per week (specific number set by individual site, goal range was 21–195 per week, varied by site size); mammography: 85% of images meeting quality criteria as assessed using a grading rubric devised and agreed by the ACR and collaborative participants; prostate: increase the percent of high‐quality images achieving a PI‐QUAL^11^ score of 4 or 5 (specific goal set by individual site, goal range was 80%–94%); RFU: appropriateness: increase the percentage of appropriate and actionable follow‐up recommendations for patients with an indeterminate lung nodule(s) (specific goal set by individual site; goal range was 45%–95%); RFU: timely follow‐up: increase the percentage with actionable recommendations for an indeterminate lung nodule(s) who receive timely follow‐up (specific goal set by individual site, goal range was 50%–75%).

^c^ Total only counts each participating site once. Recommendations follow‐up (RFU) had two measures; thus, when a site met one or both indicators, they are counted only once. One site in each of the cohorts achieved goal on both RFU indicators.

We identified four themes that explained the influence of the collaborative structure on site improvements and sustained success. These themes encompass combinations of AHRQ elements and provide explanatory value in how discrete AHRQ collaborative taxonomy elements interact to drive site performance in the collaborative. All participants felt they were successful and benefitted from the learning collaborative, even if they did not achieve their goal at graduation. Themes spanned all four of the AHRQ primary collaborative elements of *innovation*, *communication*, *time*, and *social systems* (AHRQ constructs are italicized throughout). Themes, connection to the AHRQ taxonomy elements and study specific examples and definitions, and exemplar quotes are presented in Table 4.

**TABLE 4:** Definitions of themes and connection to AHRQ taxonomy elements with exemplar quotes.

Theme	Primary AHRQ taxonomy element: Secondary AHRQ taxonomy element with study‐specific description of element	Exemplar quotes
1: Structured education in quality improvement and access to quality improvement tools provides a framework to improve	Innovation: Supporting tools Rubric to measure image quality (mammography and prostate only) Structured data collection and reporting templates provided by the ACR for reporting metric performance Varied tools for QI learning: videos, toolkit, checklists, guides, activity summary, and manuscripts or white papers provided before and during learning sessions Email templates provided by the ACR for communicating with site team members	“I think [data collection and review] is one of the biggest intakes from the quality improvement project. Now I'm like, ‘Data does show a lot of things’. Just going by that and doing my own data and trying to see ‘Is what I'm doing impacting the positioning? Is it helping?’ Things like that.” (Mammo, C2, participant 10) Our tracking was just to have the doctors tell us this information. There wasn't really a good way to do it. So this gave us that ability to, “Okay, well here's your tools. Here's what you can do. Here we can kind of go across this big scope of things.” Manually, if we had to have tried to put [the data collection tool and template] together ourselves, it would've been a lot. (Mammo, C2, participant 09) The takeaway we did get was about how to trial and error things successfully and correctly. That was a big one for us. Instead of just saying, “Okay, this is our new idea. Let's go with it,” no. There's a method to trying things, small sample size first for a small limited time first to a small amount of people first. That is the biggest takeaway that we got. (LCS, C2, participant 13) “The helpfulness was the structure on the email [template]. The email is something that […] should be read within two to three minutes and they get all the information there. It's quick, it's fast. […] So the email template that they had when we're sending out the information to our team on our progress, that was amazing. That was great.” (Mammo, C2, participant 06)
2: Expert‐guided, structured improvement process sets the pace	Innovation: Degree of prescription Sponsor‐prescribed; the ACR set the goal and structured improvement process for achieving the goal Communication: Directionality Expert to peer; the ACR primarily ran learning sessions and experts gave feedback during “walk the walls” Peer to Peer; most sessions included time for participants to share their experiences with their peers and in Cohort 1 participants were invited to share their experiences with Cohort 2 Communication: Frequency Weekly communication from the ACR facilitators to QI coaches and team leads; bi‐weekly for other team members Time: Duration of learning collaborative Collaboratives were short‐term (i.e., 6 months) with opportunities to continue longer engagement with the network	“Going through this ACR project, it showed us how to slow down, get all the data, collect it, have everyone participate and have everyone give their crazy ideas, whatever they want. And then say out of all these crazy ideas, these are the ones that we're going to try to implement and see how we can change this. And this is just a work in progress.” (Mammo, C2, participant 06) “I really particularly found learning from other institutions helpful. Just for me on a personal level just because of the way I came into the program, I didn't know anything really about lung cancer screening. And so it was actually really helpful to be part of the collaborative, to familiarize myself with the program as well as the various stakeholders involved. It was very helpful for me working collaboratively with all those people and seeing lung cancer screening as not this set thing, but something that's evolving and a work in progress and just be introduced to the people that I could collaborate with.” (LCS, C1, participant 28) “We knew we had these calls coming up, and so it forced us to all have times in our calendar. We would all sit together, we would talk about this. And having been on other projects before where you don't, I mean, we would always try and have some regular cadence, but you can always kind of postpone it or whatever. This was a national meeting and you wanted to be there. And so, I think it really helped keep us on track and keep focused.” (Prostate, C1, participant 30)
3: Intentional participant interaction and contribution in collaborative meetings reinforces accountability	Social systems: Membership characteristics Site teams were diverse, but a small number of sites participated in the collaborative and had to be successful in an application process to participate thereby likely limiting participation to those who the ACR perceived were likely to succeed in the collaborative Social systems: Purpose and degree of shared vision Goal was specified by ACR; higher agreement from mammography participants about the goal; less agreement among lung cancer screening participants about the goal for the collaborative Social systems: Members' activity level Participation in learning sessions and walk the walls was required, and common tasks had to be completed by all sites Social systems: Roles, structure, and process The ACR specified what roles at each site were needed; site teams were diverse to reflect multiple levels within the organization needed to make improvement	I think just having that support, I think was the biggest impact […] having a big support group because the LIPs, the interpreting physicians, the interpreting [radiologists …] They all were involved and they would get on some of the calls. And we would also involve them with our walk the walls and our graduation. And so, just hearing that we're all doing great, I think that was … And having that support group was one of the biggest things that made us successful. Because I came into this and I told the girls, I said, “Look, everybody's watching us. I don't want to fail.” (Mammo, C2, participant 05) “One is a quality manager of the Department of Radiology, and then they were considered our coaches. And then we also had the lead of quality over the hospital itself. And so we had some really great coaching from them. We had one of my department radiologists who was a lead, and the sponsors being the director of radiology, and the chairman of radiology/lead interpreting of breast imaging were the sponsors. So we had a lot, a lot, a lot of support. And two of the mammographers were on the team as well.” (Mammo, C2, participant 02) We didn't understand it was just [the ACR Network] were only going to look at new lung screening patients, getting the people in. We thought they were going to look at what we were good at, the annual recalls, the short term, the three‐ and six‐month follow‐ups, that kind of stuff. And when that wasn't a focus of the collaborative, we made it a focus for us and we have, I will say, succeeded in it. (LCS, C1, participant 21)
4: Credible leadership and facilitation sustains participation	Social systems: Degree of credibility of host or convener and leadership Leadership of ACR as sponsor and innovator with expert facilitators seen as essential to participation and commitment among site participants Social systems: Governance Sites had to complete an application process and sign agreement to complete tasks to be accountable	“I think just the way that they taught us and made us feel confident. Their guidance and their energy and the way that they explained everything from the very beginning of a project charter and the first step of an A3, they really walked us and held our hands through every little step and just kept reassuring us along the way. And I think just them giving us that education just makes you feel more confident and like okay, maybe this is going to work. But it really was them, like [the ACR instructor], her energy and her positivity is just off the wall. I don't know how she does it, but it comes through and it definitely helps us when we're like, oh God, I don't know if I can do this anymore. The energy and the guidance was so good for us.” (Mammo, C2, participant 12) “[ACR Network convener] comes across as really, really engaged. I think that that enthusiasm is contagious. They're good cheerleaders, but they also are good accountability partners.” (LCS, C2, participant 16)

### 3.1 Theme 1: Structured education in quality improvement and access to quality improvement tools provides a framework to improve

Participants described that a key part of their success and one of the most valuable aspects of collaborative participation was access to structured quality improvement tools and education in how to use them effectively. The education and tools provided a framework for improvement work. The collaborative taught a structured approach to quality improvement based on A3 methodology, teaching participants how to identify the root causes of their problem, implement small tests of change, and measure improvements iteratively.^12^ The quality improvement tools used weekly included A3s, process mapping, Gemba walks, Pareto charts, and Fishbone diagrams.^12^ These tools are represented in the AHRQ framework under *innovation* as the secondary element of *supporting tools*. The types of *supporting tools* that were noted as most valuable supported participants in how to measure their goals and outcomes and demonstrated the importance of data collection (*measure, data, reporting*), how to systematically conduct and document a quality improvement project (*toolkit, videos, manuscripts, guides, activity summary*), and how to communicate within a team (*templates, videos*). Some participants described that they had conducted QI projects previously, but had never applied quality improvement tools with as much rigor as they did under the collaborative structure in which they were guided by experts. While many participants noted that such a rigorous approach was worthwhile learning, some stated that they did not intend to continue to use the tools with the same fidelity after participation in the collaborative due to the time‐consuming nature of the work.

### 3.2 Theme 2: Expert‐guided, structured improvement process sets the pace

Whereas learning QI methods and how to use tools as described in theme 1 provided an essential framework for the program and a foundation for success, QI learning was reinforced through completing a project under guidance from ACR experts who set the pace for improvement efforts. Participants credited the collaborative's structure and the QI project support from the ACR for keeping sites focused and accountable. The related AHRQ taxonomy elements from *innovation, communication*, and *time* include: *degree of prescription, expert to peer directionality*, and *frequency and duration of the learning collaborative*. The clinical practice improvement was prescribed by the ACR Network for each collaborative, such as increasing lung cancer screening rates or improving breast imaging quality using a defined rubric. Communication and organization of the collaborative was primarily in the direction of the ACR Network experts to the collaborative participants, which provided structure for each site's project. The frequency of meetings was regular (weekly or biweekly) and the duration of the collaborative was short term, being 6–9 months in order to increase feasibility of participation among sites as preliminary scoping work had identified that longer periods were not likely to be feasible. Participants described that participation in the collaborative was time intensive and at times difficult to schedule time with their whole team. However, they also felt that such a demanding schedule was necessary to keep them accountable for the project and helped them achieve the improvements that they did within the collaborative timeframe. Frequent meetings with the ACR Network were also helpful for applying quality improvement methods learned in a systematic manner. While participants appreciated a shorter timeline in order to enhance overall feasibility of collaborative participation, notably, none of the lung cancer screening sites were able to achieve their goal by graduation. Interviews with cohort 1 participants indicated that it took at least 1 year for improvements to be reflected in the data due to the lag between an intervention and the time to schedule a patient.

### 3.3 Theme 3: Intentional participant interaction and contribution in collaborative meetings reinforces accountability

A defining feature of a learning collaborative is the “all teach, all learn” concept and this theme reflects the AHRQ concept of *social systems*, specifically *membership characteristics, purpose and degree of shared vision, members' activity level*, and *roles, structure, and process*. The collaboratives had dedicated time each week for cross‐organizational discussion, and the biweekly walk the walls required participants to share their ongoing work in a structured format and group setting. Participants noted high engagement from other sites and described that the “peer pressure” from presenting to other sites was a positive motivator for completing tasks. Quality improvement leaders within each organization often described feeling direct accountability within their organization for improvements and not wanting to fail their teams. Having a wide range of roles within the teams, which was required by the ACR network as part of their participation, particularly having leadership support, was described as an important feature of a site's success.

Participants also appreciated hearing how similar sites were approaching their improvement efforts and sharing common struggles. The importance of shared experiences was particularly notable because of the network format with four collaboratives that interacted during learning sessions. Participants cited that sharing experiences with those in the same collaborative and with similar organizational structures (e.g., size, insurance model) was often of most value. These shared experiences also helped participants to make sense of the outcome measures used by the collaborative. Some of the lung cancer screening cohort in particular were concerned about whether and how they would define their eligible population (i.e., the denominator group) to assess whether they were increasing screening volumes; therefore, some sites felt that the collaborative goal was not perfectly aligned with their own goals. However, hearing how other clinics were approaching the same issues helped sites to clarify what was most important to them in their improvement efforts that still aligned with the overall vision for the collaborative.

### 3.4 Theme 4: Credible leadership and facilitation sustains participation

The fourth theme also related to the AHRQ element of *social systems*, specifically *degree of credibility of host or convener and leadership*, and *governance*. The learning network and collaboratives had a clear *governance* structure that established the learning collaborative roles and responsibilities, rules of engagement, operation, and accountability. Sites also had to apply to participate to ensure that they could adhere to the collaborative structure and process. Some participants described that they had applied to participate in cohort 1 but were unsuccessful, and therefore were highly committed to their participation in cohort 2. The collaborative was governed by a steering committee from the ACR with advisory committee support that was comprised of experts in the various fields of radiology. Participants acknowledged that the leadership of the ACR, which is a regulatory body and trusted organization, was a primary factor in deciding to join the collaborative and lent the collaborative a high *degree of credibility*. The individual experts who provided content expertise for each collaborative were also perceived to be knowledgeable, insightful, and helpful in their problem‐solving. The collaborative facilitator helped to motivate participants by setting expectations, encouraging learning and growth, and supporting reflection. Credible and inspirational leadership engendered a sense of trust and belief in the educational materials, and buy‐in to the ideas being presented, allowing participants to “trust the process” and sustained their participation in the collaborative.

## 4 DISCUSSION

This study takes a novel approach at examining how learning collaboratives can drive health care improvements and can provide guidance to collaborative designers. We identified four distinct themes that explained how the discrete elements of a learning collaborative helped organizations successfully conduct quality improvement projects and achieve improvements in care in a radiology setting. These themes encompassed a range of AHRQ taxonomy collaborative elements that spanned all four primary elements (*innovation, communication, time*, and *social systems*). An important contribution of this study is connecting the elements of a learning collaborative to actions or mechanisms that explain improvements observed at participating sites. A learning collaborative is one strategy to conduct systematic quality improvement and implement practice changes.^1^, ^13^, ^14^, ^15^ It can therefore be difficult to disentangle the impact of the learning collaborative from the quality improvement methods used during participation in the collaborative. Indeed, the ACR learning network and collaboratives were intentionally highly structured to facilitate QI learning through doing.^9^ Though not all collaboratives promote specific quality improvement methods and yet still achieve improvements,^7^ our observation from this study is that there is likely a synergistic relationship between the two: a high‐quality learning collaborative structure supports and is reinforced by a reliable and rigorous quality improvement process. Participation in a highly structured collaborative can act as a strategy that forces adherence to systematic quality improvement methods. This is evident by the four themes identified which simultaneously reflect the collaborative structure, as defined using AHRQ elements, and key features of structured process improvement.^16^

Additionally, most participants described sustained changes in their approach to improvement at a site level, such as the ongoing integration of data into decision making, that was prompted by participation in the collaborative, suggesting that the collaborative may have helped to build local learning communities.^17^ This was particularly important for the lung cancer screening cohorts as this was the only collaborative in which the length of the collaborative, or *time* element, did not align with the outcomes of interest (screening volumes). As compared to prostate and mammography collaboratives in which interventions to improve image quality could be immediately assessed, interventions to increase screening rates took longer periods to reveal their impact. It was therefore important for participants to continue collecting data and monitoring performance to assess cycles of improvement efforts beyond graduation from the collaborative. This suggests that the *time* element is important on at least two dimensions that must be balanced: sustaining engagement from individuals who have to return to their usual day‐to‐day work at their site at some point, and allowing sufficient time for interventions to have impact on quality measures.

While we identified all four primary elements of the AHRQ framework as important for a successful learning collaborative, two of our themes represented only *social system* elements, suggesting the prominence of these AHRQ elements for implementation success. *Social system* elements have been previously identified as important influences on success, such as the normative pressure for accountability created by peer interaction.^3^, ^18^, ^19^, ^20^ Our observations and interviews suggested a crucial element of success that is not explicitly represented in the AHRQ framework, nor is it often well‐defined in the quality improvement and implementation science literature: each of the learning sessions and walk the walls was led by an external leader who participants described as a “cheerleader.” As one person put it: “her energy and her positivity is just off the wall.” Leadership, particularly through coaching, is critical for successful collective learning, though often this is conceptualized as internal organizational leadership.^3^, ^19^, ^21^ In this case, the ACR learning network leadership was critical to engaging teams and keeping them accountable, in part because of their perceived expertise in the field, but also because of the interpersonal connections and leadership styles. Pacesetting and coaching leadership styles were identified during collaborative meetings.^22^ Our findings suggest that these soft skills, and even charisma, may impact success as much as, if not more, than structural components of a learning collaborative. Participant success was reflected in their engagement and feelings of accountability, which were mainly influenced by aspects of the *social system*, especially the *credibility of the convener and leadership* and the overall supportive *culture of the collaborative*.

Coding of transcripts spanned the AHRQ primary elements (*innovation, communication, time social systems*) and we found that some of the AHRQ secondary elements (e.g., *degree of credibility of host/convener* and *governance, expert to peer directionality* and *degree of prescription*) were interconnected. Though each element has been separately defined, there were related concepts that could be better understood together rather than as stand‐alone independent features; this is evident in our theme descriptions which encompass multiple AHRQ elements. Similar to previous studies that have applied the AHRQ framework as an analytic tool, we found that it was useful as a guiding framework to help describe the collaborative, though some concepts, such as *scope* or *culture of collaborative*, were vaguely or incompletely defined.^23^ Many secondary elements were defined with discrete categories, such as *members' activity level* within *social systems* is defined as either “active” or “passive,” which makes it possible to categorize collaborative features. However, *culture of learning collaborative* is defined with more abstract concepts, such as “degree of trust, understanding, and respect; flexibility; participation; vested interests in process and outcomes.”^8,p. 210^ More concrete definitions of these secondary elements would help facilitate comparison of features across collaborative structures. Our thematic groupings, which connect collaborative elements to processes and outcomes, may offer collaborative designers a framework of elements to focus on for designing successful collaboratives.

Our study had several limitations. First, not all participants who participated in the collaborative agreed to be interviewed; therefore, there is likely some bias in the responses from those who were active or had positive experiences. Our analysis focused on collaborative structure contributors of success, and as most sites experienced some success, we were not able to significantly assess instances where sites were not successful. From the limited follow‐up information we received from sites that did not see significant improvement, lack of improvement was often due to site‐specific issues, such as staffing or site leadership changes that limited their ability to participate. Due to resource constraints, we were only able to observe walk the walls for two of the four collaboratives for cohort 2, though during learning sessions, participants from all four collaboratives were present. The focus on radiology‐specific collaboratives may limit the transferability of findings to collaboratives in other specialty settings. Future research should explore collaboratives in other clinical settings using the AHRQ taxonomy to assess whether the predominant elements and relationships between elements identified for success in this study emerge in other settings.

## 5 CONCLUSION

Learning collaboratives represent a powerful tool for implementing practice improvements in healthcare by leveraging collective expertise and fostering collaborative problem‐solving. A highly structured approach, as leveraged by the ACR learning network, has the potential to drive significant advancements in patient care. Key to learning collaborative success was expert, credible leaders who provided the right tools at the right time and pace, with constructive guidance. The communal learning environment fostered a healthy sense of peer pressure and accountability for sites to maintain momentum throughout their projects. This study demonstrates the important influence of social systems and a dynamic approach to leadership as a key feature of learning collaborative success.

## AUTHOR CONTRIBUTIONS

Laura M. Holdsworth, Marcy Winget, and David Larson conceived of and designed the evaluation. Laura M. Holdsworth, Heather Z. Mui, and Kay Zacharias‐Andrews collected and analyzed the data. Laura M. Holdsworth and Heather Z. Mui drafted the manuscript. All authors reviewed and approved the final manuscript.

## CONFLICT OF INTEREST STATEMENT

The authors declare no conflicts of interest.

## Supporting information


Data S1.
