# Supplementary material for: Evaluation of four learning collaboratives for improving diagnostic excellence in radiology
Source: Learn Health Syst. 2025 Aug 22;9(4):e70035. doi: 10.1002/lrh2.70035 (PMC12569447; doi:10.1002/lrh2.70035)
Supplement: Supplementary file 1 — Data S1. [file LRH2-9-e70035-s001.docx]

Topic guide for Cohort 2 participants

| Main question | Prompts/Probes |
| --- | --- |
| **Background: I’d like to start with a little bit of background information about yourself.** | |
| First, could you please tell me a bit about your role? | Clinicians: When did you complete your training?  What you enjoy most about your job?  Did you participate in Cohort 1? |
| FIRST Interview (with QI coach/leader):  Can you tell me more about your team that participated in the collaborative? | Number of radiologists  Techs  Managers, others?  How was it decided who should participate in the project? |
| **Achievements: Thanks for that information. I now want to talk about what you feel your center has achieved from when you first started participating in the collaborative until now.** | |
| Can you start by telling me why your organization wanted to join the collaborative? | What was the culture of QI/learning in your organization before joining the collaborative? Has it shifted now?  What did you learn about QI that you will continue to use in the future, and why?  Have you participated in a collaborative before? |
| What were some things that went really well? | Why do you think those things went well? |
| What were some things that didn’t go so well? | Were you able to overcome those challenges? How? |
| Do you feel like you achieved what you were hoping to achieve for your center by the time of graduation? | What do you think was key to your success? (leadership, time) *OR* What barriers did you encounter in reaching those goals? |
| **ACR collaborative structure: Thanks for that information. I’d now like to talk about the structure of the learning collaborative and how it helped you to make the changes you have** | |
| Did you participate in:   - Project scoping meeting (project charter, overview of roles, expectations and boundaries) - Measurement discussions - Learning Sessions - Walk the Wall - Other? | What did you think about them?  What did you find helpful?  What was not as helpful? |
| Overall, what did you think about the format of collaborative? | What was the most useful part of the collaborative?  Did you learn from other sites? What did you learn?  How useful was it to talk to people in other collaboratives in small groups?  How useful was it to have people who had been in the previous cohort presenting their stories at Walk the Wall? |
| **Lessons learned: Lastly, I’d like to talk about your future plans for sustaining improvement and lessons learned.** | |
| What do you expect to continue doing that you were doing while in the collaborative? | What things are you going to stop doing or change? Why? |
| What advice would you give to other centers who are thinking about participating in an ACR Network Collaborative? | What things would a center have to have in place or be willing to do to be successful in the collaborative? |
| What would you suggest changing for Cohort 3? | Is there anything else ACR could do to support sites?  What are your thoughts on the incentives (CME credits, star recognition, etc.)? What other incentives would be motivating? |
| Is there something else you’d like to tell me about that I haven’t asked about? |  |

Topic guide for interviews with Cohort 1 participants

| Main question | Prompts/Probes |
| --- | --- |
| **Background: I’d like to start with a little bit of background information about yourself.** | |
| First, could you please tell me a bit about your role? | What do you enjoy most about your job? |
| Can you tell me more about your team that participated in the collaborative, and your role? | Number of radiologists, techs, managers, others? |
| **Lessons learned: Thank you for sharing that information. I now want to talk about what you learned from participating in the collaborative and ACR network.** | |
| What was the most valuable thing you got out of the collaborative?  (QI process/ skills/ tools; interventions/ workflow/ built in process to help improve [positioning / increase volumes / image quality / appropriate follow-up]) | What supporting tools were most helpful and valuable? (measure/instrument, data collection and reporting, tools to support QI learning: videos and checklists)  What about the data/measure/tools was so valuable? What were you doing/ using/ measuring/ collecting before the collaborative? How was it different than the collaborative?  What about the structure (virtual meetings, coaching calls, dedicated website, and emails) and frequency? |
| How did the collaborative help you to make improvements? | Do you think you would have been able to make these improvements without being in the collaborative? Why or why not?  What was different in working through a collaborative to improve, compared to trying to do it yourself/on your own?  Have you tried to make improvements before? How did it go? |
| *For sites who met their goal during the collaborative*: Have you been able to sustain the improvements you achieved during the collaborative? | IF YES: How have you been able to sustain those achievements?  IF NO: What have been the barriers to sustaining those gains?  Have you continued to implement the interventions that helped you to make improvements? Why or why not? Have you adapted those processes/interventions in order to sustain them over time? |
| *For sites that didn’t meet goal:* Were you able to eventually reach your goal? | IF YES: What helped you to eventually reach your goal?  IF NO: What challenges have you encountered in trying to achieve that goal?  Are you still trying to meet goal? If not, why not? |
| In terms of process improvement methods, what are you continuing to do, if anything, that you learned or implemented during the collaborative? | Are you collecting and using data to make decisions?  Are you working with different people to make decisions/ consensus building?  Creating shared mental models and improving communication? |
| Are you applying PI/QI methods to other departments or projects? How and why? | Are you using certain principles or specific tools or formats (or going through the same structured course with a different project/goal)? |
| **Continued ACR Network participation: Thank you for that information. I’d now like to talk about the network structure and participation after ImPower program.** | |
| Are you continuing to participate in the quarterly meetings? | Do you feel like you have collaboration opportunities with people from other cohorts?  How much collaboration is there with participants from other cohorts?  How much ongoing collaboration is there with participants from your own cohort? |
| Do you have ongoing support from the collaborative (from ACR collaborative leaders or peer organizations who participated)? | If so, for what? How does that help you?  What ongoing support from the ACR are you getting?  If not, why not? |
| What additional support would be helpful? |  |
| Is there something else you’d like to tell me about that I haven’t asked about? |  |
